# An Event-Related Potential Study on the Effects of Cannabis on Emotion Processing

**DOI:** 10.1371/journal.pone.0149764

**Published:** 2016-02-29

**Authors:** Lucy J. Troup, Stephanie Bastidas, Maia T. Nguyen, Jeremy A. Andrzejewski, Matthew Bowers, Jason S. Nomi

**Affiliations:** 1 Department of Psychology, Colorado State University, Fort Collins, Colorado, United States of America; 2 Department of Psychology, University of Miami, Coral Gables, Miami, Florida, United States of America; University of Tasmania, AUSTRALIA

## Abstract

The effect of cannabis on emotional processing was investigated using event-related potential paradigms (ERPs). ERPs associated with emotional processing of cannabis users, and non-using controls, were recorded and compared during an implicit and explicit emotional expression recognition and empathy task. Comparisons in P3 component mean amplitudes were made between cannabis users and controls. Results showed a significant decrease in the P3 amplitude in cannabis users compared to controls. Specifically, cannabis users showed reduced P3 amplitudes for implicit compared to explicit processing over centro-parietal sites which reversed, and was enhanced, at fronto-central sites. Cannabis users also showed a decreased P3 to happy faces, with an increase to angry faces, compared to controls. These effects appear to increase with those participants that self-reported the highest levels of cannabis consumption. Those cannabis users with the greatest consumption rates showed the largest P3 deficits for explicit processing and negative emotions. These data suggest that there is a complex relationship between cannabis consumption and emotion processing that appears to be modulated by attention.

## Introduction

### Emotion Processing

There are a variety of explanations of how the brain processes emotion emphasizing differing levels at which an explanation is focused. Some approaches emphasize a physiological structural account, others are based on a higher, more “cognitive” level of understanding, with less emphasis on the underlying structures [1, 2, 3]. A recent meta-analysis of a decade’s worth of data addressed two possible accounts for how emotion is processed in the brain: a “locationalist” account, where a specific brain location is responsible for eliciting a particular emotion, and a “psychological constructionist” account, which suggests that processing of emotion is distributed across brain structures [4]. Recent research investigating the temporal processing of emotion suggests that early processing of emotional stimuli, measured electrophysiologically, were modulated by task [5,6, 7]. Rellecke et al asked participants to either, explicitly identify the emotional expression of a face stimulus, or implicitly process emotional expression in a passive viewing task. When participants were presented with faces expressing varying emotions (angry, happy and neutral) early ERP components such as the P1 and N170 were affected by task requirements. Angry expressions elicited a larger P1 and N170 than happy or neutral expressions in both implicit and explicit tasks, whereas happy expressions elicited higher amplitudes compared to neutral in a later time window of 200–600ms [5]. It appears then that implicit/explicit processing interacted with emotional expression such that angry expressions influenced early components regardless of tasks, while happy expressions only influenced later components when explicitly attended. This suggests that emotion processing is best explained by emotion specific differences in attention and best fits with a constructivist description of emotion processing [4, 5, 6, 7].

### Emotion Processing and Cannabis

The effects of cannabis on cognition and the brain is a rapidly evolving area of investigation which has provided evidence for a complex interaction between physiological and psychological processes. Cannabis consumption elicits both immediate (acute), residual and long-term changes in brain activity, that are manifested throughout the body such as altered appetite and food intake, altered sleep patterns, and changes in measures of executive function and emotional behavior [8]. Crean et al reviewed studies reporting acute, residual and long term effects of cannabis in adults from a Medline and Psych Info search. Acute effects being defined as 0–6 hours post consumption, residual effects being defined as 7–20 hours post consumption and long term effects being 3 weeks or longer post consumption. [8]. A summary of acute effects includes impairment in decision making, increase in risk taking and attentional deficits, which are most pronounced in casual users. Residual effects appear to be focused on executive function whilst long term effects are difficult to establish from the literature [8]. Many factors influence how cannabis use affects information processing. There is conflicting evidence as to the effects that cannabis has on mood states and emotional processing in the brain, especially considering the role of the endocannabinoid system in stress responses and overall emotional states [9, 10]. For example, it has been suggested that cannabis consumption increases both positive and negative mood states [10]. Differences in emotion type and amount of cannabis consumed are also of interest. For example Ballard et al. reported a dose-dependent relationship in chronic users between the amounts of Δ^9^-tetrahydrocannabinol (THC) consumed and participants’ ability to identify emotional expressions in faces showing negative emotions such as fear and anger, but had little effect on faces showing sadness and happiness [11]. Behavioral data from a recent functional magnetic imaging (fMRI) study also suggests that cannabis users have the most deficits compared to controls in response to negative emotions in expression recognition tasks. Participants who had been given cannabis prior to the study showed poorer performance on a face matching task for negative (fearful) emotional faces compared to positive (happy) emotional faces [12]. In a recent study addressing emotional expression recognition, using a four-way double-blind, placebo-controlled, crossover design, the acute administration of cannabis-derived THC suppressed emotional expression recognition. Participants were shown faces portraying six basic emotions (happiness, sadness, anger, disgust, fearful, surprise and neutral) which were morphed from 10% to 100% of the expression. Each emotion was then presented as 5 levels of intensity (20%, 40%, 60%, 80% and 100%) for each emotion. Participants had to identify the emotion presented to them in a recognition paradigm. Accuracy of recognition of emotional expression was impaired by the administration of THC, but performance was improved by the subsequent administration of cannabidiol (CBD). This suppression effect that THC has can then be resolved through the endocannabinoid system specifically by administering CBD, one of the many endocannabinoid derivatives found in cannabis [13]. This suggests that the effects of cannabis on affective processes are complex. Deficits in recognition of emotional faces seem to be weighted toward negative emotions, and the magnitude of these deficits also appears to be related to specific cannabinoid receptors suggesting that strain of cannabis is important to the effects it might have on emotion.

Behavioral studies have also linked heavy cannabis use to impairments in emotion processing compared to controls. In a dynamic emotional expression task where participants were asked to identify emotional expressions as faces morphed from open mouthed to an expression, either positive (happy) or negative (fearful) cannabis user’s accuracy and reaction time performance was impaired. Individuals who used cannabis fifteen times a month and more than fifty times in their lifetime showed increased reaction times and decreased accuracy to the faces that became negative [14].

Recent research seeking to clarify the effects of cannabis use on brain structure is mixed. In particular, structural magnetic resonance imaging (MRI) data indicated no significant differences in the amygdala, a critical structure for emotion processing, in both adult and adolescent brains of daily users [15]. However, research also suggests that the use of THC can alter brain structures and facilitate change in regions recruited during emotion processing. A recent structural study using both human and nonhuman subjects suggested that cannabis does have a detrimental effect on the brain structures associated with emotional processing, significantly changing the size and gray matter density of nucleus accumbens and amygdala [16]. Although this study specifically addressed the addictive nature of the drug, the structures that appear to be damaged in respect to THC are also heavily implicated in emotional processing in the brain. Whilst this is an important study, structural deficits are not always reflected in functional deficits in neurophysiology. Cognitive processing and brain function are not always isomorphically linked. A meta-analysis conducted by Lindquist et al looked at how robust structural accounts of emotion are [4]. They concluded that there is little evidence that discrete emotions can be attributed to activity in particular brain structures. This suggests that a specific emotion is not necessarily processed by a particular discrete brain area, and the evidence to support structural accounts of emotion processing is limited. This then provides strong support for “psychological constructionist” accounts of emotion processing. Suggesting that emotion processing is distributed over interacting networks in brain, rather than processed in discrete regions [4]. Further research is clearly needed on the effects of cannabis on emotion processing

### Emotion and Event Related Potentials

One approach that can be considered a valuable tool in further investigating the complexity of emotion processing is event related potential (ERP) methodologies. Structural changes in the brain do not always translate to differences in function and behavior. EEG techniques allow us to investigate the relationship between biomarkers (brain mechanisms) of a distributed neural network and associated behavior. This approach is particularly relevant to determine whether or not cannabis use has an effect on the circuitry of the brain when structural differences have not been found.

EEG recordings provide information with resolution in the milliseconds. This approach consists of the measurement of the summated firing of neurons in the cortex through electrodes placed on the scalp. When this activity is averaged and time-locked to a specific event (e.g., presentation of an image of a happy face), an event-related potential (ERP) is obtained. ERPs provide an indication of the temporal dynamics of cortical activity following that specific event, allowing us to obtain information on the time course of emotion processing. This average is then compared to those obtained for other conditions (e.g., showing an image of a sad face) to examine whether processing of different types of information diverges in time and across electrode sites. Through extensive research some patterns in ERPs associated with cognitive processes have been characterized by ERP components—the amplitude of the waveform at a specific point in time. For example, the P3 or P3 complex component is defined as a change in voltage in a positive direction (increase in amplitude) occurring between 200–400ms on average after stimulus onset [17].

One ERP in particular is of interest to emotion processing and also cannabis use. The P3 complex component has been associated with task-relevant stimulus evaluation and attention allocation and it is has been consistently linked to emotion processing [18, 19, 20]. Specifically, Johnston et al showed that there is a greater P3 amplitude elicited by emotional (positive and negative) stimuli, compared to neutral stimuli, during passive viewing and emotion discrimination tasks [21].This effect is most noticeable over right electrode sites compared to a more symmetrical P3 during an emotionally-neutral comparison task [21, 22, 23]. During oddball presentations of emotional stimuli, P3 amplitudes are enhanced for unpleasant stimuli compared to pleasant and neutral stimuli, over posterior electrode sites. P3 amplitudes present similar increases for images with greater arousal values, independent of valence [24]. Interestingly the effects of emotion on the P3 appear to be modulated by task demands [25]. Krolak-Salmon et al asked participants to focus attention on emotion by identifying the emotional expression in a face data set. In a second condition participants attention was not directed to emotion rather they were asked to make a gender identification judgment with the same stimulus set. Late latency ERPs were significantly different when attention was directed to emotion than when it was not [25]. In a recent review of the ERPs related to emotional processing Hajcak et al discuss the emerging importance of the P3 as a significant marker in emotion processing [26]. They go on to emphasize the role of attention in emotion processing and the P3 suggesting that there are both automated and control driven processes that effect the ERPs associated with emotion processing [26].

The exact nature of the P3 and its relationship to emotion is still very much under investigation [27]. However, there is a body of research linking the P3 with a variety of psychiatric disorders including mood disorders [28]. In a single trial analysis study of depression a reduced P3 was shown to be a marker for negative mood [29]. P3 effects associated with processing of threatening faces have also been identified in individuals with anxiety disorders, underscoring the use of ERPs as measures of altered processing of emotion associated with changes in brain function of endogenous and exogenous origin [29].

### Event Related Potentials (ERPs) and Cannabis

Cannabis use has been associated with deficits in the P3 especially pertaining to complex cognitive tasks such as working memory, and selective attention [30, 31, 32]. Böcker et al measured the acute effects of THC exposure on visual attention. Participants were asked to smoke cannabis in four separate doses, over four days, ranging from low to high levels of THC, two hours before performing an attention task. Prior exposure rates for their participants included a use pattern of 6–18 cannabis cigarettes a month (median of 8) and a mean prior length of exposure to cannabis being 6.5 years. The P3 component was significantly affected by dose, with a reduction in amplitude occurring as THC dose increased [30]. Theunissen et al also investigated acute cannabis exposure compared to occasional use in a P3 paradigm. Heavy cannabis use was defined as more than four time a week, occasional users smoked less than twice a week. All of their participants reported using cannabis in conjunction with tobacco. As with the previous study levels of intoxication corresponded with a decrease in the P3 component whilst performing an attention task [31]. In an auditory attention task Kempel et al showed similar deficits in attentional processing with those who reported early onset exposure having the greatest deficits in the P3 [32]. D’Souza et al demonstrated a dose-dependent modulation of the P3 amplitude in a within-subjects study measuring performance pre- and post-cannabis administration in an oddball paradigm: the greater the dose, the smaller the P3 amplitude [33]. There were no effects on latency in the P3. This reduction in the P3 in an oddball paradigm was also observed in a comprehensive neuropsychological and neuropharmacological study looking at the effects of various common drugs on the P3 [34].The effects of cannabis on casual users and current non-users with a history of cannabis use are less clear. Solowij et al. presented a case study that demonstrated a smaller P3 in cannabis users with at least five years prior exposure history, and at least 6 weeks abstinence prior to testing, that showed a partial recovery to pre-cannabis use amplitudes after 6 weeks of abstinence in an auditory attention task [35].

The consistent P3 pattern exhibited during emotional processing represents an effective means of assessing the effects of cannabis use on neural processing. The P3 has implications for understanding both the short and long-term effects cannabis use has on emotion processing. Despite this the majority of research into the effects of cannabis on emotional expression recognition has focused on either behavioral approaches, for example Ballard et al [11], or structural imaging techniques, for example the work of Hindoca et al [13]. There has also been less emphasis on the effects of casual use of cannabis on cognitive processing and specifically emotion processing. This is of particular interest in states where recreational cannabis use is legalized and there is a significant increase in casual users because of its new legal status.

### Current Study

To better understand the role of attention on the effects of cannabis in emotion processing our current study uses a paradigm based on Relleke et al [5] and extended in our lab to include an empathy condition. [6, 7]. The addition of an empathy condition allows assessment of an individual’s ability to consciously identify and relate to emotion in others in later processing. At the same time it is possible to observe how tasks that do not require directed attention (implicit condition) differ from those that do (explicit and empathic conditions). By manipulating task demands based on previous research [5, 6, 7] we would expect to see differences in the P3 in both cannabis users and controls dependent on whether they are explicitly directing their attention to a particular emotional expression compared to when the attentional demands of the task direct them away from emotional expression recognition in the implicit processing task. If cannabis modulates attention, we would also expect to see greater differences in cannabis users compared to controls. This would be greatest in the empathic condition where attention to emotion is fully engaged. We also seek to clarify the role of both negative and positive emotional valence in emotional expression recognition in cannabis users compared to controls in a P3 paradigm. Of particular interest in our study is the effect of recreational cannabis use on emotion processing. This approach is limited as it is impossible to control for the type of endocannabinoid and potency in a recreational user model. It is also difficult to control for other confounding variables such as acute compared to residual effects of cannabis. However we are interested in the effects of cannabis as it is used in a real world recreational setting and feel that is important to investigate cannabis effects in such an ecologically valid model of use. This is of particular importance with the legalization of cannabis for recreational use in several states including Colorado. It is also important with a growing body of research suggesting that it is a drug that is used extensively to self-medicate for mood disorders with both positive and negative outcomes [36].

If cannabis use effects emotion processing we would expect to see differences in the P3 to our emotional stimuli for our cannabis user group compared to controls. If attention is also affected by cannabis use, then greater demands on attention, driven by the levels of attentional engagement, elicited by our three processing tasks, implicit, explicit and empathic would also lead to P3 differences.

We therefore hypothesize that cannabis users will show a difference in P3 amplitude compared to non-cannabis using controls. This will be further influenced by the type of emotion and task demands.

## Methods

### Participants

Seventy-three undergraduate students and volunteers recruited from the community provided written consent and self-report of demographic information which is summarized in Table 1. Behavioral data reported for the empathy task excluded two control and one cannabis user due to missing data. Final participant number reported was then seventy. Forty three control participants who had never used, or had used cannabis minimally in the past (more than one year past, see Table 2. For details of participant use information), and twenty seven self-reported current cannabis users with reported use ranging from casual to chronic exposure. Participants were screened for current prescription medications, caffeine intake, and tobacco, cannabis, alcohol, and other drug use in the last 8 hours and 24 hours. History of exposure to other substances was minimal. Only four participants reported using tobacco with a range of 1–4 cigarettes in 24 hours. Self-reported alcohol use was minimal with four participants reporting using alcohol but with no use in the last 8 hours, and a range of 1–5 beers in the last 24 hours. Only one participant reported using both alcohol and tobacco. They were also screened for significant mental health issues including personal and family history for mood related disorders and drug related disorders, and concussion and head injury. No significant impairments including neurological and visual deficits were reported.

**Table 1:** Summary of reported participant demographic and neurological/behavioral assessment, showing mean scores and ranges as well as scoring profiles for the empathy and depression scales used. Significant group differences at α = .05 are shown.

*Factor*	Control (n = 43)	User (n = 27)	*F*	df	*p*	Cohen’s *d*
Female *n* (%)	32 (74.4)	19 (70.4)				
Right-Handed *n* (%)	38 (88)	21 (77.8)				
Age	19.3±2.07 (18–28)	21.3±7.18 (18–47)	2.935	1,69	.092	-.43
Depression Scores ^a^	15.3±6.88 (4–38)	16.1±6.89 (5–27)	-.210	1,63	.648	-.12
State Anxiety Scores ^b^	33.3±9.56 (21–54)	32.5±10.6 (22–57)	.101	1,62	.752	.06
*Empathy Scores* ^c^						
Fantasy Scale	19.1±4.46 (10–27)	19.3±4.07 (10–26)	-.028	1,63	.867	-.05
Perspective-Taking	23.4±3.89 (15–31)	23.7±4.96 (14–33)	-.078	1,63	.781	-.07
Empathic Concern	24.1±4.86 (14–32)	25.3±3.77 (17–31)	-1.176	1,63	.282	-.27
Personal Distress	16.2±5.6 (7–26)	15.3±5.31 (5–25)	.405	1,63	.527	.17

Group means are presented as Mean ± Standard Deviation (Range).

^a^Center for Epidemiological Studies-Depression Scale (CES-D). Scores on this scale range from 0 to 60.

^b^ State portion of State-Trait Anxiety Inventory (STAI).

^c^ Interpersonal Reactivity Index (IRI).

**Table 2:** Summary of cannabis use reported on the Recreational Cannabis Use Evaluation (R-CUE), showing group frequencies and percentages for each response option.

Cannabis Use Factor	Control (n = 43)	User (n = 27)	Overall (n = 70)
*Years Since First Use n (%)*			
N/A (Never Tried)	35 (81.4)	0 (0.0)	35 (50.0)
< 1 year	0 (0.0)	6 (22.2)	6 (8.6)
1–2 years	1 (2.3)	8 (29.6)	9 (12.9)
2–4 years	2 (4.6)	4 (14.8)	6 (8.6)
4–7 years	2 (4.6)	5 (18.5)	7 (10.0)
7–10 years	2 (4.6)	1 (3.7)	3 (4.3)
10+ years	1 (2.3)	3 (11.1)	4 (5.7)
*Current Frequency of Use n (%)*			
NA (Don’t Use)	43 (100.0)	0 (0.0)	43 (61.4)
1–11x per Year	0 (0.0)	18 (66.7)	18 (25.7)
1–3x per Month	0 (0.0)	2 (7.4)	2 (2.9)
1–2x per Week	0 (0.0)	1 (3.7)	1 (1.4)
3–6x per Week	0 (0.0)	1 (3.7)	1 (1.4)
1x per Day	0 (0.0)	2 (7.4)	2 (2.9)
2–4x per Day	0 (0.0)	3 (11.1)	3 (4.3)
More than 4x per Day	0 (0.0)	0 (0.0)	0 (0.0)
*Past Frequency of Use n (%)*			
NA (Never Used)	39 (100.0)	0 (0.0)	39 (55.7)
1–11x per Year	0 (0.0)	13 (48.1)	13 (18.6)
1–3x per Month	0 (0.0)	2 (7.4)	2 (2.9)
1–2x per Week	3 (0.0)	1 (3.7)	4 (5.8)
3–6x per Week	1 (0.0)	3 (11.1)	4 (5.8)
1x per Day	0 (0.0)	2 (7.4)	2 (2.9)
2–4x per Day	0 (0.0)	4 (14.8)	4 (5.8)
More than 4x per Day	0 (0.0)	2 (7.4)	2 (2.9)
*Reported Intake Methods n (%)*			
Flower	0 (0.0)	24 (88.9)	24 (34.3)
Concentrates	0 (0.0)	9 (33.3)	9 (12.9)
Edibles	0 (0.0)	9 (33.3)	9 (12.9)
Dermal	0 (0.0)	0 (0.0)	0 (0.0)

Undergraduate students who were recruited from the departmental research pool received credit in a Psychology course for their participation. Participants from the local community received no compensation. The study was approved by Colorado State University’s Office of Research Integrity & Compliance Review Office Institutional Review Board (IRB) Protocol ID: 12-3716H.

### General Procedure

All participants provided written consent and completed a general demographic questionnaire. They were further screened for symptoms of depression and anxiety using the Center for Epidemiological Studies Depression Scale (CES-D) [37] and the State portion of the State-Trait Anxiety Inventory (STAI) [38]. Cutoffs for excluding participants from analysis were 16 or more for CES-D, 35 or more for STAI, consistent with norms associated with these tests. Participants also completed a measure of self-reported dispositional empathy (Interpersonal Reactivity Index) [39] in four separate but related dimensions: perspective-taking, fantasy, empathic concern, and personal distress (See Table 1 above for summary).

After the screening and assessment portion of the study was completed, participants were fitted with a recording EEG cap, detailed below in the EEG acquisition portion of the methods. During the recording, detailed below, participants completed an emotion processing task, presented on a Dell desktop computer at a viewing distance of 30cm using Stim2 software [40, 41].

**Fig 1:**
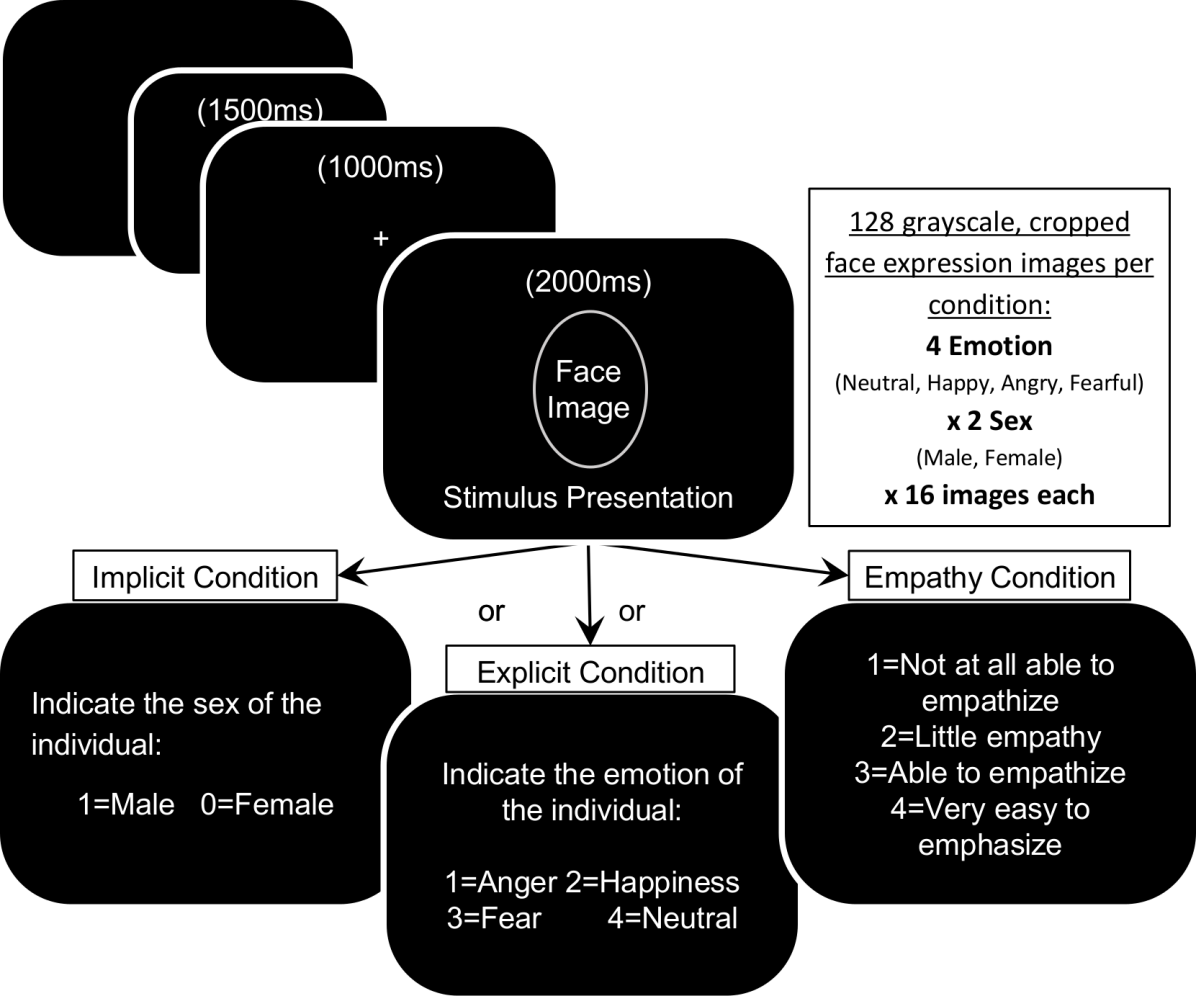
Summary of Experimental Design. Each block began with detailed instructions for the condition to be completed. Participants were reminded before each facial expression to identify the sex, emotional expression, or own ability to empathize with the depicted expression using the ratings shown. All completed each of the three conditions in a randomized order, counterbalanced across participants.

The emotion processing task required that participants viewed faces depicting positive (happy), neutral, and negative (angry and fearful) emotional expressions, obtained from the Radboud Faces Database [42]. Thirty-four novel faces (17 female) with expressions of anger, happiness, fear, and neutral were edited to transparent background, gray-scale, and resized to 320x390 pixels using ImageMagick software. Stimuli were fully randomized into three blocks of 128 trials (32 unique faces x 4 expressions each), one block for each task condition, and their order was counterbalanced in order across participants. There were also eight practice trials (2 unique faces x 4 expressions each) at the beginning of each block. There was an inter stimulus interval of 1500ms followed by a fixation cross presented for 1000ms. Face stimuli then appeared for 2000ms followed by the instruction response screen which differed dependent on task condition (see Fig 1 for summary). Participants were instructed to respond as accurately as possible and were given 2000ms to respond. The three task conditions were comprised of an explicit emotional processing task, where the participants were asked to identify the emotion that was presented; an implicit emotional processing task, where they had to identify the sex of the face, male or female; and an empathy task where they were asked to empathize with the emotion shown and rate their ability to empathize. Instructions for the task to be completed were presented at the beginning of each block. A prompt before each image presentation reminded participants of the instructions to be followed, after the face expression was viewed, when they were asked to press a key to indicate the sex, emotion, or rated ability to empathize for the previous stimulus.

After EEG data collection, cannabis use was assessed by self-report using a questionnaire developed specifically for this study; The Recreational Cannabis Use Evaluation (R-CUE). R-CUE was created to better understand the ecology of cannabis use in Colorado’s recreational system of high volume, potency, and variety. Thus, R-CUE consists of questions regarding type of use and method of intake—including use of edibles, concentrates, and transdermal applications—in addition to information about potential current use, relational previous use, and years of use. Based on participants’ responses they were assigned to control or cannabis user groups (see Table 2). Cannabis users were further classified as casual, if they reported use of once a week or less (n = 20), or chronic (n = 7) for those reporting a frequency of use greater than once per week (see Table 2). This is consistent with previous research categorizing users into heavy and recreational users [43]. The methods used in this study have been used previously in studies examining emotion processing [6, 7].All of our cannabis users fit the legal cannabis use requirements under Colorado State law, meaning they were over the age of 18 with a medical cannabis card or over the age of 21 for recreational users. The average age of our chronic user group was 21.85 years with 6 out of the 7 chronic users being between 18–20 years old. The average age of our casual user group was 20.5 years of age.

### EEG Acquisition

Electroencephalogram (EEG) was recorded from 25 Ag/AgCl electrodes covering regions of interest (ROI’s) consistent with the measurement of the P3 ERP component and identified based on previous research [6, 7, 41] (midline: Fz, Cz, Pz; left: Fp1, F3, F7, FC1, C3, T7, CP1, P3, P7, PO7, O1; and corresponding right electrodes) mounted on a SynAmps2 64-channel QuikCap [41] according to the 10–20 system [44]. The ground electrode being midline anterior to Fz and online reference placed at the right mastoid. Signals were recorded at a sampling rate of 500Hz and amplified with a band pass of .10–50Hz in epochs from -200 to 1000ms. Horizontal electro-oculogram was monitored with electrodes placed on the outer canthi of the left and right eyes [45]. Due to limitations presented by the use of the QuickCap system vertical electro-oculogram was recorded using electrodes FP1 and FP2 and recordings from these electrodes were included in our artifact rejection. Impedance was kept below 5kOhm.

### Data Analysis

Data analysis was divided into two parts. Firstly the analysis of behavioral responses where average reaction times and percent correct responses during each task were calculated for all participants. The second part was the analysis of the ERPs derived from the EEG data collected during the behavioral task. Behavioral responses were scored for all trials based on reaction time (RT) in milliseconds, percent correct for sex and emotion identification tasks, and average rated empathy (1–4 scale) for the empathic task. Participants with over 90% "no responses" on any of the three task conditions were excluded from analysis, as were individual trials with RTs faster than 100ms. Repeated measures analyses of variance were performed by cannabis use grouping (control, user) and emotion (neutral, happy, angry, and fearful) for RT, accuracy, and rated empathy scores during each task.

EEG was re-referenced offline to the common average and baseline corrected to pre-stimulus interval of 200ms. Artifact rejection was applied using the built in artifact rejection tool in SCAN 4.5 EEG acquisition software [41]. Trials exceeding amplitudes of ±100μV at any electrode, and participants for whom all trials for any one condition were rejected (e.g., all empathy rating trials for happy faces) were excluded from analysis. P3 mean amplitudes were calculated in the 200–400ms interval and compared in repeated measures analyses of variance by cannabis use grouping (control, user) by task (implicit, explicit, empathic) by emotion (neutral, happy, angry, fearful) by hemisphere (left, right) measured over ROIs based on previous research, and included parieto-occipital (PO7/8), parietal (P3/4), centro-parietal (CP1/2), central (C3/4), and fronto-central (FC1/2) sites. These sites were chosen as most representative of the general pattern of activity corresponding to the P3 component. All statistical analyses included Greenhouse-Geisser corrections to violations of sphericity when appropriate, and follow-up *t*-tests on significant differences with α = 0.05 for planned group comparisons and Bonferroni correction for post-hoc tests where appropriate. Eta-squared measures of effect size were reported for all within-group factors, while Cohen's *d* was reported for between-group effects.

## Results

Overall differences in behavioral measures were consistent with differences in ERPs in relation to task, and emotion. This suggests that ERP differences were driven by cannabis exposure and not by differences in responses to emotion or task. A detailed description of the results are as follows.

### Behavioral Results

**Fig 2:**
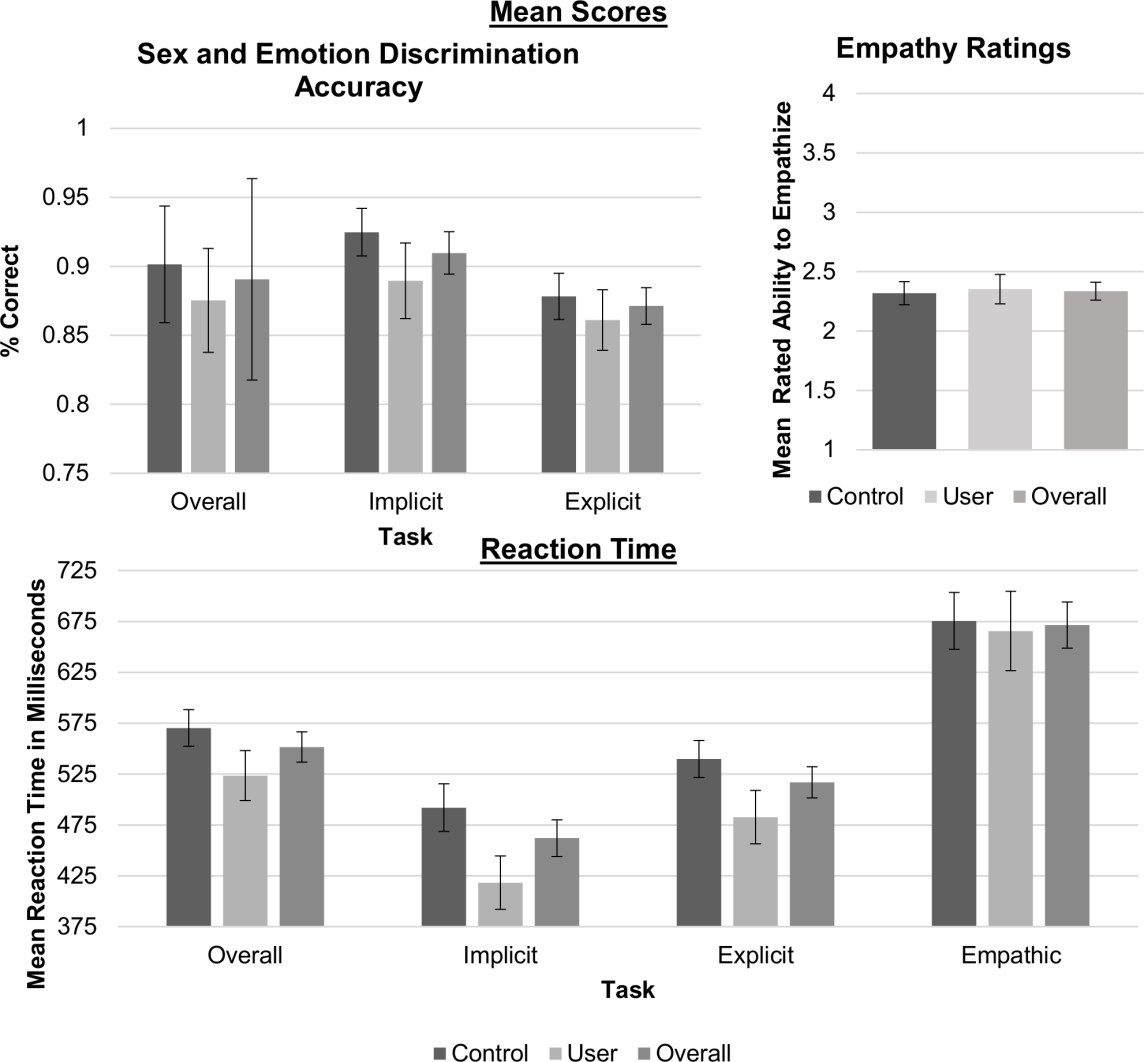
Performance for implicit, explicit and empathy tasks between groups in % correct and reaction time in milliseconds with error bars representing SEM.

**Fig 3:**
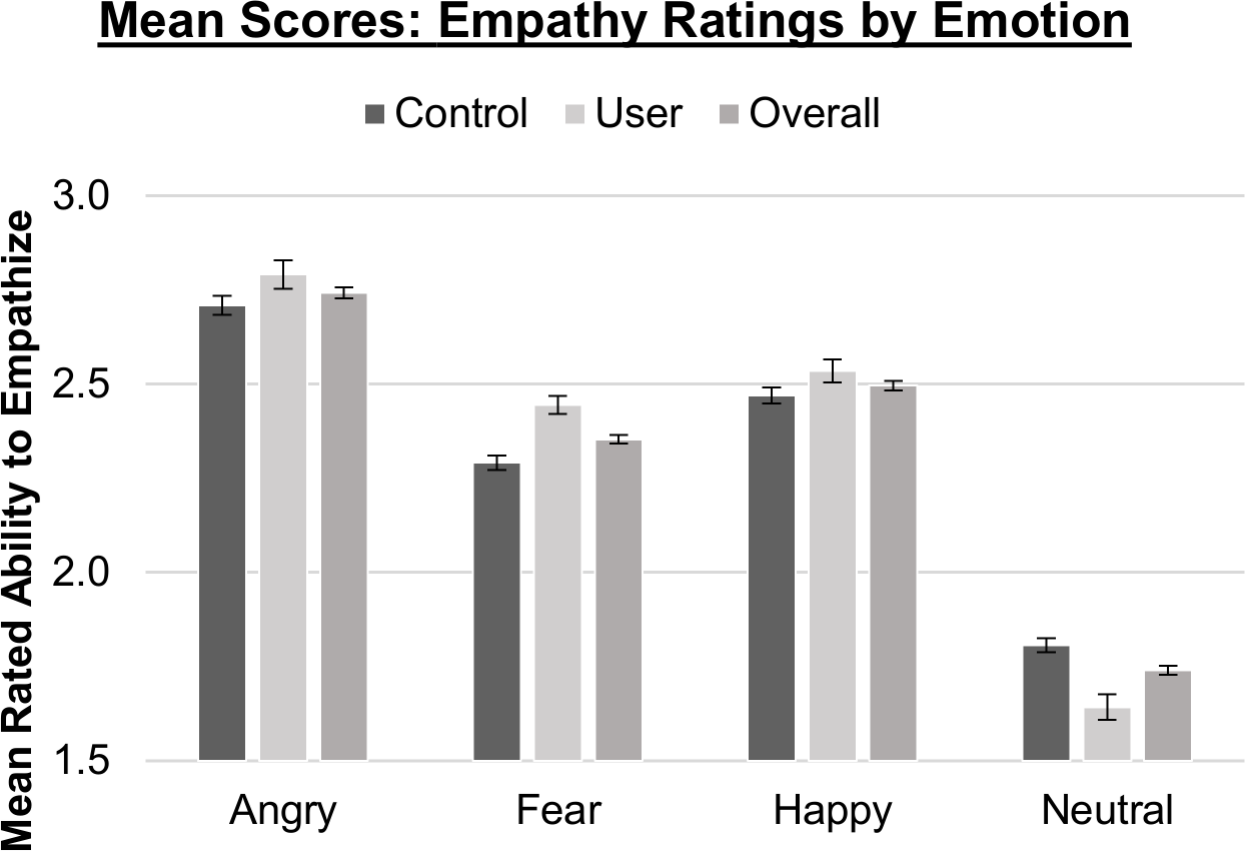
Mean empathy ratings for all four emotional expressions, neutral, happy, angry & fearful with error bars representing SEM.

In the emotion processing task we examined the effect of cannabis use grouping and emotional expression on participants’ reaction time (RT) in milliseconds and response scores in a repeated measures analysis of variance (see Table 3 for group means). We calculated percent correct scores for sex identification (implicit condition), and emotion identification (explicit condition), and average rated ability to empathize (1–4 scale, empathic condition) for each trial. While overall differences were found between task conditions and emotional expressions there were no significant main effects or interactions for cannabis use on performance (see Tables 3 & 4 for a summary of results). That is, cannabis users’ RT and response scores did not differ from controls’, and both groups presented the same result patterns during implicit, explicit, and empathic tasks (see Figs 2 & 3).

**Table 3:** Mean Reaction Times and Response Scores for Emotion Processing Tasks. Effect Sizes for Group Differences are also shown.

Factor	Control (n = 39)	User (n = 26)	Cohen’s *d*
*Reaction Time (ms)*	570.3±112.92 (358–856)	523.5±125.37 (333–758)	.40
Sex ID	492±144.46 (257–860)	418.3±134.06 (197–707)	.53
Emotion ID	539.8±113.55 (317–869)	482.7±134.23 (277–790)	.48
Empathy Rating	675.5±174.87 (395–1010)	665.6±198.52 (328–1073)	.05
*Response Scores*			
Sex ID Accuracy (%)	0.92±0.1 (0–1)	0.89±0.14 (0–1)	.26
Emotion ID Accuracy (%)	0.88±0.1 (0–1)	0.86±0.11 (1–1)	.20
Empathy Ratings (1–4)	2.3±0.6 (1–3)	2.4±0.63 (1–3)	-.17

**Table 4:** Statistical Analysis of Reaction Time and Response Scores Differences by Group and Emotion for Emotion Processing Tasks.

	Reaction Time				Response Scores			
Factors	*F*	df	*p*	ηp^2^	*F*	df	*p*	ηp^2^
*Task*: *Implicit (Sex ID)*								
*Within-Group*								
Emotion	2.613	3,171	.053	.044	1.553	3,186	.202	.024
Stimulus Sex	**11.977**	**1,57**	**.001**	**.174**	.327	1,62	.570	.005
Emotion * Stimulus Sex	.584	3,171	.627	.010	1.913	3,186	.129	.030
*Between-Group*								
Group	2.591	1,57	.113	.043	.400	1,62	.529	.006
* Emotion	1.697	3,171	.169	.029	.724	3,186	.539	.012
* Stimulus Sex	2.402	1,57	.127	.040	1.232	1,62	.271	.019
* Emotion * Stimulus Sex	.617	3,171	.605	.011	.216	3,186	.885	.003
Task: Explicit (Emotion ID)								
*Within-Group*								
Emotion	**73.751**	**3,183**	**< .001**	**.547**	**13.185**	**3,186**	**< .001**	**.175**
Stimulus Sex	.014	1,61	.905	.000	2.087	1,62	.154	.033
Emotion * Stimulus Sex	2.488	3,183	.065	.039	**4.513**	**3,186**	**.005**	**.068**
*Between-Group*								
Group	2.893	1,61	.094	.045	.396	1,62	.531	.006
* Emotion	2.224	3,183	.096	.035	1.730	3,186	.175	.027
* Stimulus Sex	.761	1,61	.386	.012	.497	1,62	.483	.008
* Emotion * Stimulus Sex	.200	3,183	.887	.003	1.251	3,186	.293	.020
Task: Empathic (Empathy Ratings)								
*Within-Group*								
Emotion	**12.633**	**3,186**	**< .001**	**.169**	**34.554**	**3,186**	**< .001**	**.358**
Stimulus Sex	.036	1,62	.850	.001	4.106	1,62	.047	.062
Emotion * Stimulus Sex	.127	3,186	.944	.002	2.079	3,186	.104	.032
*Between-Group*								
Group	.046	1,62	.831		.048	1,62	.828	.001
* Emotion	.649	3,186	.573	.010	.860	3,186	.452	.005
* Stimulus Sex	.010	1,62	.922	.000	.151	1,62	.699	.002
* Emotion * Stimulus Sex	2.198	3,186	.090	.034	.328	3,186	.784	.014

#### Reaction Time

A significant effect of sex on RT for implicit processing was characterized by slower RT for sex identification of female compared to male faces, *F*(1,57) = 11.977, *p* = .001. Emotional expressions did not differ in RT during implicit processing, but they did have a significant effect on RT for explicit and empathic responses. Participants’ responses were the slowest for emotion identification of neutral expressions (angry: *t*(63) = -8.785, fearful: *t*(63) = -7.401; all *p*s < .001), with similar RTs for angry and fearful expressions, *t*(63) = -1.772, *p* = .081, and the fastest for happy expressions (vs. neutral: *t*(63) = -5.120, angry: *t*(63) = -11.532, fearful: *t*(63) = -12.379; all *p*s < .001). Empathic rating scores also differed by emotion, with slower RTs for neutral than angry expressions, *t*(63) = -2.814, *p* = .007, and slowest for happy compared to negative expressions (vs. angry: *t*(63) = -8.785, fearful: *t*(63) = -7.401, *p*s < .001), with a similar trend compared to neutral, *t*(63) = -2.447, *p* = .017. RTs for fearful expressions during the empathy task did not differ from neutral, *t*(63) = -1.751, *p* = .085, or angry expressions, *t*(63) = -1.735, *p* = .088.

#### Response Scores

A main effect of emotion (neutral, happy, angry, and fearful expression) on response scores was significant for explicit and empathic tasks (*p*s < .05): emotion identification accuracy scores were lowest for angry faces (vs. happy: *t*(63) = 6.838, *p* < .001; neutral: *t*(63) = 3.209, *p* = .002; fearful: *t*(63) = 5.933, *p<001*), lower for neutral than fearful faces (*t*(63) = -2.700, *p* = .009), and no other differences for happy faces (vs. neutral: *t*(63) = 1.149, *p* = .255; fearful: *t*(63) = -.849, *p* = .399). Further, a two-way interaction of emotion by sex indicated greater accuracy for male angry faces than female angry faces, *t*(63) = 2.965, *p* = .004, *d* = .27. The empathy task was characterized by lowest rated ability to empathize for neutral expressions (vs. happy: *t*(63) = -8.997, angry: *t*(63) = -5.882, fearful: *t*(63) = -6.640; all *p*s < .001), higher ratings for happy than angry expressions, *t*(63) = 3.733, *p* < .001, and no other differences for fearful expressions (vs. happy: *t*(63) = 2.193, *p* = .032; angry: 2.182, *p* = .033). A main effect of sex on empathy ratings suggested slightly higher rated ability to empathize with female than male faces, independent of emotion, *F*(1,62) = 4.106, *p* = .047. There were no significant effects of emotion or sex on response scores for implicit trials.

### P3 ERPs

**Fig 4:**
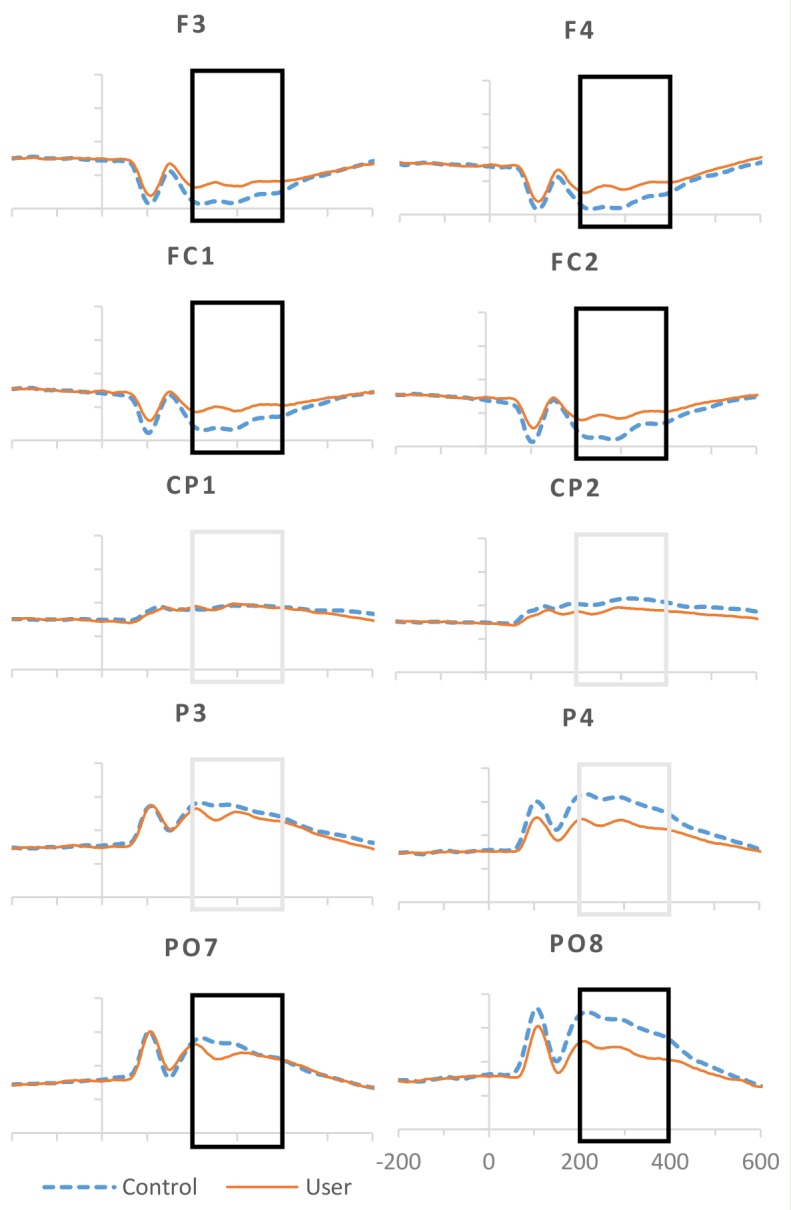
Grand average ERPs for controls and users collapsed over task and emotion for analyzed electrodes. Significant group differences at α = .05 with Bonferroni correction for planned multiple comparisons are signaled in black.

Mean P3 amplitudes calculated at five ROIs, and analyzed in a repeated measures analysis of variance, identified significant main effects of cannabis use grouping, task instructions, and emotional expression, and interactions among these factors (see Table 5 for P3 amplitude means and Table 6 for detailed statistical results). Participants with all trials rejected for any single condition were excluded from analysis. This resulted in only participants with 75% of trials remaining. Grand average P3 amplitudes differed by electrode site, with maximal amplitudes at parieto-occipital (PO7/PO8) sites, followed by parietal (P3/P4), frontal (F3/F4), fronto-central (FC1/FC2), and centro-parietal sites (CP1/CP2), *F*(1,68) = 80.518, *p = 001*, *ηp*^2^ = .542 (see Fig 4). No main effects of hemisphere on amplitude were observed.

**Table 5:** Averaged P3 Amplitudes (in microvolts) of Controls and Cannabis Users from Emotion Processing of Neutral, Happy, Angry, and Fearful Expressions.

Factors	PO7/PO8		P3/P4		CP1/CP2		FC1/FC2		F3/F4	
	*Control*	*User*	Control	*User*	*Control*	User	*Control*	*User*	Control	User
*Implicit*	0.874 (1.7)	0.628 (1.4)	-2.557 (2.5)	-1.468 (1.6)	-2.386 (2.2)	-1.151 (1.1)	2.843 (3.4)	1.751 (2.1)	3.288 (4.3)	1.472 (2.2)
Angry	0.94 (1.6)	0.504 (1.9)	-2.282 (2.2)	-1.331 (1.5)	-2.245 (2.1)	-1.091 (1.1)	2.686 (3.1)	1.666 (2.1)	2.93 (4.3)	1.407 (2.0)
Fearful	0.91 (1.7)	0.558 (1.3)	-2.583 (2.4)	-1.494 (1.5)	-2.305 (2.0)	-1.185 (1.0)	2.884 (3.5)	1.716 (2.2)	3.332 (4.0)	1.477 (2.1)
Happy	0.888 (1.8)	0.695 (1.5)	-2.604 (2.6)	-1.344 (1.5)	-2.328 (2.1)	-0.993 (1.1)	2.855 (3.3)	1.594 (2.1)	3.394 (4.2)	1.168 (2.1)
Neutral	0.758 (1.9)	0.755 (1.2)	-2.76 (2.9)	-1.704 (1.7)	-2.665 (2.4)	-1.333 (1.2)	2.946 (3.6)	2.026 (2.2)	3.498 (4.6)	1.837 (2.5)
*Explicit*	1.101 (1.4)	0.938 (1.4)	-2.62 (2.0)	-1.228 (1.3)	-2.21 (1.8)	-0.998 (1.0)	2.847 (2.9)	1.712 (1.7)	2.943 (3.2)	1.281 (2.1)
Angry	1.079 (1.4)	0.967 (1.4)	-2.499 (2.0)	-1.253 (1.3)	-2.164 (1.8)	-0.891 (1.0)	2.694 (2.8)	1.678 (1.6)	2.67 (3.0)	1.175 (2.3)
Fearful	1.136 (1.5)	0.938 (1.3)	-2.517 (2.0)	-1.257 (1.3)	-2.153 (1.8)	-0.995 (1.0)	2.818 (2.8)	1.757 (1.7)	2.865 (3.2)	1.292 (1.9)
Happy	1.063 (1.5)	0.981 (1.5)	-2.652 (2.0)	-1.145 (1.3)	-2.144 (1.8)	-0.906 (1.0)	2.851 (2.7)	1.693 (1.7)	3.125 (3.3)	1.082 (2.1)
Neutral	1.125 (1.4)	0.868 (1.2)	-2.812 (2.0)	-1.256 (1.3)	-2.378 (1.8)	-1.198 (1.2)	3.024 (3.1)	1.719 (1.7)	3.112 (3.3)	1.574 (2.1)
*Empathic*	0.949 (1.6)	0.902 (1.4)	-2.391 (2.1)	-1.309 (1.5)	-2.135 (1.9)	-1.009 (1.0)	2.776 (3.1)	1.715 (1.8)	2.874 (3.5)	1.353 (2.2)
Angry	0.942 (1.6)	0.783 (1.5)	-2.205 (2.0)	-1.267 (1.6)	-2.122 (1.9)	-1.105 (1.2)	2.64 (3.1)	1.727 (1.8)	2.795 (3.5)	1.402 (2.3)
Fearful	1.031 (1.7)	0.818 (1.2)	-2.48 (2.0)	-1.219 (1.7)	-2.047 (1.8)	-0.888 (1.0)	2.823 (2.9)	1.605 (1.7)	2.81 (3.1)	1.139 (2.1)
Happy	0.881 (1.5)	0.984 (1.6)	-2.564 (2.1)	-1.453 (1.4)	-2.338 (1.9)	-1.075 (1.0)	2.841 (3.0)	1.754 (1.9)	3.139 (3.5)	1.367 (2.2)
Neutral	0.94 (1.7)	1.023 (1.2)	-2.315 (2.3)	-1.298 (1.3)	-2.033 (1.9)	-0.969 (0.9)	2.799 (3.3)	1.775 (1.7)	2.752 (3.9)	1.502 (2.3)

**Table 6:** Statistical Analysis of P3 Mean Amplitudes across Electrode Sites.

Factors	PO7/PO8			P3/P4			CP1/CP2			FC1/FC2			F3/F4			
	*F*	*p*	ηp^2^	*F*	*p*	ηp^2^	*F*	*p*	ηp^2^	*F*	*p*	ηp^2^	*F*	*p*	ηp^2^	df
*Within-Group*																
Task	2.30	.10	.0	.15	.87	.0	**5.52**	**.01**	**.1**	**3.93**	**.02**	**.1**	1.43	.24	.0	2,136
Emotion	**4.28**	**.01**	**.1**	**3.63**	**.01**	**.1**	.29	.84	.0	**4.01**	**.01**	**.1**	**3.04**	**.03**	**.0**	3,204
Hemisphere	2.06	.16	.0	.19	.66	.0	2.55	.12	.0	1.38	.25	.0	.92	.34	.0	1,68
Task * Emotion	1.42	.21	.0	.57	.76	.0	.20	.98	.0	**3.82**	**.00**	**.1**	1.32	.25	.0	6,408
Task * Hemisphere	1.99	.14	.0	.20	.82	.0	2.15	.12	.0	.37	.70	.0	1.80	.17	.0	2,136
Emotion * Hemisphere	.39	.76	.0	.32	.81	.0	2.48	.06	.0	.96	.41	.0	1.20	.31	.0	3,204
Task * Emotion * Hemisphere	.66	.68	.0	.24	.96	.0	1.62	.14	.0	.67	.67	.0	1.26	.28	.0	6,408
*Between-Group*																
Group	**8.36**	**.01**	**.1**	**4.99**	**.03**	**.1**	.32	.58	.0	**11.59**	**.00**	**.1**	**8.74**	**.00**	**.1**	1,68
* Task	.52	.59	.0	.08	.93	.0	.75	.48	.0	.30	.75	.0	1.69	.19	.0	2,136
* Emotion	**3.89**	**.01**	**.1**	.94	.42	.0	1.92	.13	.0	.65	.59	.0	.97	.41	.0	3,204
* Hemisphere	.01	.91	.0	.04	.83	.0	.01	.92	.0	.31	.58	.0	.00	1.00	.0	1,68
* Task * Emotion	.23	.97	.0	.68	.67	.0	.74	.62	.0	.45	.85	.0	.40	.88	.0	6,408
* Task * Hemisphere	**3.19**	**.04**	**.0**	.17	.85	.0	.58	.56	.0	1.29	.28	.0	.43	.65	.0	2,136
* Emotion * Hemisphere	.78	.51	.0	.46	.71	.0	.43	.73	.0	.20	.89	.0	.62	.60	.0	3,204
* Task * Emotion * Hemisphere	.94	.47	.0	.68	.67	.0	.32	.93	.0	.35	.91	.0	.32	.93	.0	6,408

Bonferroni correction for multiple comparisons was applied across electrode site measurements (corrected *α* = .01) and for planned follow-up comparisons (corrected *α* = .004).

#### Group Differences

**Fig 5:**
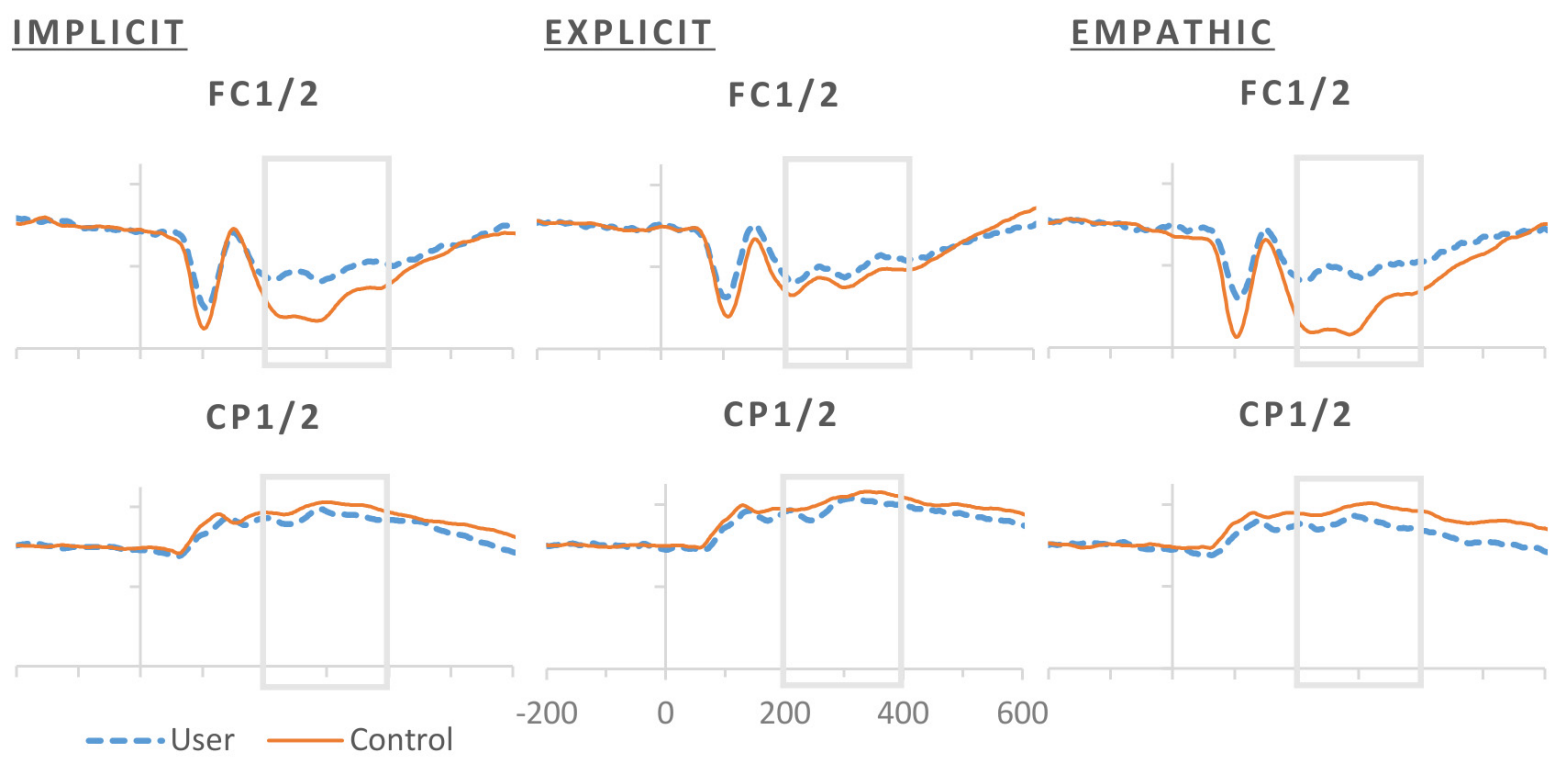
Averaged ERP responses for non-users and users for the four different stimulus emotions, happy, neutral, angry & fearful for representative electrode PO8.

Cannabis users presented smaller P3 than controls over frontal, *t*(66) = 3.498, *p* = .001, Cohen’s *d* = -.74, fronto-central, *t*(68) = 3.937, *p <* .*001*, *d* = -.56, and parieto-occipital sites *t*(62) = 4.201, *p <* .*001*, *d* = -.93, but not at parietal, *t*(68) = 2.233, *p* = .029, *d* = .20, or centro-parietal sites, *t*(68) = .955, *p* = .343, *d* = .56 (see Fig 4). An interaction of group by emotion at parieto-occipital sites suggested that users’ P3 was smaller than controls’ in response to happy (*t*(65) = -3.992, *p <* .*001*, *d* = .86) and fearful expressions (*t*(67) = -3.449, *p* = .001, *d* = .76), with a similar trend of smaller P3 for angry expressions (*t*(68) = 2.546, *p* = .013, *d* = .58) but no group differences for neutral expressions, *t*(68) = 2.347, *p* = .022, *d* = .63 (see Fig 5 for ERPs and S1 Fig for overall P3 amplitudes). Also over parieto-occipital sites, an interaction by task by hemisphere approaching significance specified this P3 difference in users to be present over left sites for implicit, *t*(62) = 3.289, *p* = .002, *d* = .70, and explicit, *t*(67) = 3.251, *p* = .002, *d* = .72, but not empathic processing, *t*(68) = 2.046, *p* = .045, *d* = .51; while right sites showed no group differences (implicit: *t*(68) = 1.622, *p* = .109, *d* = .40, explicit: *t*(68) = 2.283, *p* = .026, *d* = -.40, empathic: *t*(68) = 2.192, *p* = .032, *d* = .55).

#### Task Instructions

**Fig 6:**
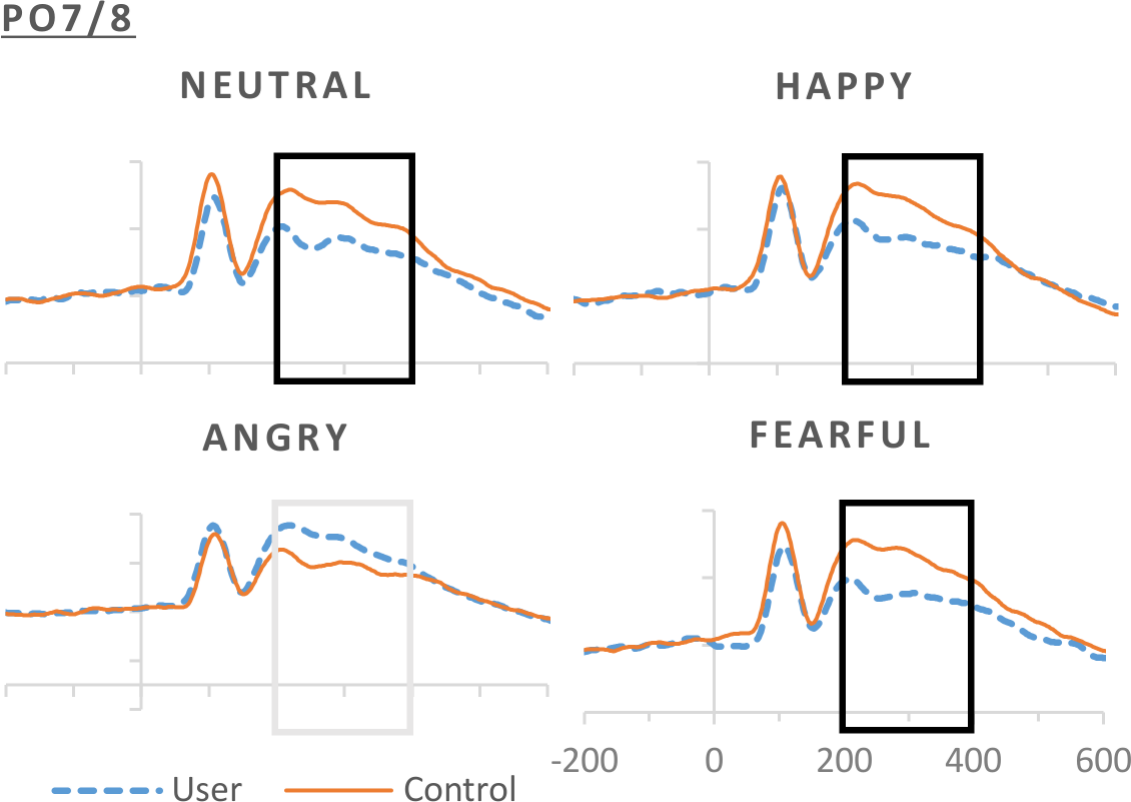
Averaged ERP responses for controls and users by explicit, implicit and empathic conditions for representative fronto-central and centro-parietal electrodes. Significant between-group differences at α = .05 with Bonferroni correction for planned multiple comparisons are signaled in black.

A main effect of task instructions was characterized over centro-parietal sites as a reduction in P3 during implicit compared to explicit processing of facial expressions, *t*(69) = -3.151, *p* = .002. In contrast, P3 at fronto-central sites was enhanced for implicit compared to explicit, *t*(69) = -2.430, *p* = .018, and empathic processing, *t*(69) = -2.430, *p* = .018 (see Fig 6). Fronto-central sites also presented an interaction of task by emotion indicating greater P3 amplitude specifically for implicit vs. empathic processing of neutral, *t*(69) = -3.880, *p <* .*001*, and fearful, *t*(69) = -3.142, *p* = .002, expressions, with no amplitude differences present for angry, *t*(69) = -.560, *p* = .577, or happy expressions, *t*(69) = .328, *p* = .744.

#### Emotion

P3 differences between expressions were observed across all but centro-parietal electrode sites (*p*s < .01): neutral expressions elicited a larger P3 than fearful (fronto-central: *t*(69) = -3.482, *p* = .001) and angry (frontal: *t*(69) = -3.055, fronto-central: *t*(69) = -3.099, parietal: *t*(69) = -3.578, parieto-occipital: *t*(69) = 4.540; all *p*s < .003), but not happy expressions (happy-angry: *t*(69) = -.766, *p* = .446; happy-fearful: *t*(69) = -.954, *p* = .343; angry-fearful: *t*(69) = -.133, *p* = .895). At fronto-central sites, these effects were modulated by a task by emotion interaction, such that during implicit processing, P3 amplitude for happy and angry expressions was reduced compared to neutral expressions (happy: *t*(69) = -3.436, *p* = .001; angry: *t*(69) = -3.296; *p* = .002), with a trend for fearful compared to neutral (*t*(69) = -2.807, *p* = .006), and no differences between emotional expressions (happy-angry: *t*(69) = -.128, *p* = .899; happy-fearful: *t*(69) = -.694, *p* = .490; angry-fearful: *t*(69) = -.758, *p* = .451). Explicit processing presented a similar reduction only for angry expressions compared to neutral, *t*(69) = -2.730, *p* = .008, with smaller P3 for happy and fearful expressions compared to neutral approaching significance (happy: *t*(69) = -2.552, *p* = .013; fearful: *t*(69) = -2.387, *p* = .020), and no other significant differences (happy-angry: *t*(69) = .089, *p* = .899; happy-fearful: *t*(69) = .524, *p* = .490; angry-fearful: *t*(69) = 1.150, *p* = .451). Effects of emotion on P3 for the empathy task were not significant after correction for multiple comparisons, *F*(3,207) = 3.352, *p* = .023. However, there was a trend suggesting smaller P3 for neutral compared to happy expressions, *t*(69) = 2.322, *p* = .023, and also a smaller P3 for happy compared to angry expressions, *t*(69) = -1.328, *p* = .008, while no other comparisons approached significance.

#### Cannabis Use Frequency

To expand on the patterns found in P3 amplitude effects of cannabis use, we examined differences within the cannabis users group based on frequency of use. For this purpose, we divided participants in this group into casual users (yearly or monthly cannabis use; *N* = 20) and chronic users (weekly or daily cannabis use; *N* = 7), and compared P3 amplitudes between the two groups and controls (no current cannabis use; *N* = 43). In this manner, significant group effects for cannabis use (control, user) were further examined using one-way analyses of variance with follow-up *t*-tests to explore the possible role of cannabis use frequency in this relationship.

For overall P3 amplitudes, group effects remained significant across the same electrode sites (*F*s(2,69): frontal = 4.645, *p* = .013, ηp^2^ = .122; fronto-central = 5.808, *p* = .005, ηp^2^ = .148; parieto-occipital = 4.505, *p* = .015, ηp^2^ = .119) with no group effects at parietal, *F*(2,69) = 2.868, *p* = .064, ηp^2^ = .079, or centro-parietal sites, *F*(2,69) = .777, *p* = .464, ηp^2^ = .023. However, no comparisons between the three frequency use groups reached significance. Next, we compared mean P3 amplitude between groups at parieto-occipital sites for each emotional expression and found significant effects of reduced P3 amplitude for happy and fearful expressions (*F*s(2,67) = 6.121 & 5.416, *p*s = .004 & .007, *ηp*^2^ = .073), but not for neutral, *F*(2,67) = 2.887, *p* = .063, or angry expressions, *F*(2,67) = 3.699, *p* = .030. Specifically, there was a trend for reduced P3 amplitude in both casual and chronic users compared to controls for happy and fearful expressions, with no differences between casual and chronic users and no differences for neutral and angry expressions.

## Discussion

### General Discussion of Results

Behavioral performance between our cannabis users and controls presented similar patterns of response. Reaction time (RT) responses were affected by explicit and empathic processing in respect to neutral and negative emotions, with angry and fearful faces giving rise to slower RT responses than happy faces in the explicit processing condition. This effect was reversed in the empathy condition where happy faces gave rise to the slowest RT responses. A similar pattern of response was observed in accuracy ratings with angry faces producing the lowest response scores in explicit and empathic processing. There were however no between group differences with cannabis users presenting the same pattern of responses as non-cannabis users. Unlike previous studies [5] we saw at greatest levels of attentional demand, namely empathic processing, higher empathy ratings for negative emotions than positive emotions. Rellecke et al concluded that when attentional engagement is maximized in their explicit processing task happy emotional expressions elicit the greatest response. However Rellecke et al did not include an empathy condition in their study.

Significant differences in ERPs were observed for group (cannabis users compared to controls), for task (implicit, explicit and empathic), and for emotion (neutral, happy, fearful and angry). Specifically, the P3 was significantly reduced in mean amplitude in our emotion processing paradigm for those participants that used cannabis compared to participants who did not. Happy expression eliciting the largest P3, followed by fear and angry expressions in users compared to controls. Differences were further modulated by task: in the implicit and empathic task conditions cannabis had a significant decrease in the P3 compared to controls when processing emotional expression. However when attention was directed in the explicit processing task they have similar P3 responses to emotional expression as controls. When comparing levels of cannabis exposure as a possible factor in our cannabis use group it appears that those who use cannabis casually have greater deficits in emotion processing with a reduced P3 response generally for all emotion conditions. This suggests that possibly increased exposure in our chronic group may have developed compensatory mechanisms to the effects on cannabis on emotion processing. However it is important to acknowledge that acute, residual and long term exposure as well as the specific endocannabinoids and other confounding compounds such as alcohol and tobacco were not specifically controlled for a priori in this study.

The dissociation between performance on behavioral measures and corresponding P3 amplitude data are interesting. One possible explanation is that exposure to cannabis alters the way in which the brain allocates resources during emotion processing as measured by the P3 component [5, 18, 19, 20], supporting a “psychological constructivist” account [4] as a more likely explanation of emotion processing.

#### Cannabis exposure

Our results are consistent with other studies investigating the effects of cannabis on the P3 as a marker for a number of cognitive processes, especially attention based tasks. Cannabis exposure has been shown to give rise to a marked decrease in the P3 component in a number of attentional tasks both visual and auditory [30, 31, 32, 33]. The reduction we observe in our data in P3, particularly in frontal electrode sites, fits with the previous literature in relation to cannabis use and its role in modulating attention marked by a decrease in the P3 as THC levels increase [31, 31] and attention in emotion processing [5, 25]. We also observe differences in the pattern of P3 effects of cannabis specifically in relation to emotion. These differences are addressed below. We also see a decrease in the P3 in emotion processing related to frequency of cannabis use with our data showing statistical trends nearing significance. In our data there are trends approaching significance for differences between participants that report using cannabis less frequently, casual users, and those that report using more frequently, chronic users. This supports previous research suggesting that THC has a dose-dependent relationship on attentional processing [31, 33]. Both Theunissen et al [31] and D,Souza et al [33] showed that as THC dose increases the P3 in attentional processing tasks decreased. In particular we observe deficits in P3 amplitude for emotion is maintained in both our casual and chronic user groups compared to controls, although a statistically significant difference between groups is not observed. This result reflects an inconsistency in the cannabis and emotion literature, where cannabis exposure both increases and decreases positive and negative emotional states [9, 10]. It is worth noting that these studies focus on the acute effects of cannabis whereas our casual and chronic user group’s cannabis use pattern is more consistent with residual or long term effects of cannabis.

#### Task

Task driven differences in the P3 were apparent in our data for both cannabis users and control conditions. With a reduction in the P3 for implicit processing compared to explicit and empathic processing in both groups. This is consistent with previous work by Rellecke et al where implicit and explicit processing of emotional expression gave rise to different patterns of ERP [5]. Our data differed in that differences we observed in later ERP components (P3) were driven mostly by negative valence emotion processed explicitly whereas Rellecke et al saw differences in the later ERP components in explicitly processed positive valence emotions [5].

Our implicit, explicit and empathic tasks were sensitive to cannabis exposure, as marked by a decrease in the P3, which is consistent with the literature indicating that visual [30, 31] and auditory [32] attention is impaired by cannabis use. However, the decrease we observe appears to be more pronounced in the implicit task and empathic tasks for cannabis users. Interestingly differences we observe in task on P3 amplitude is not consistent across scalp locations. Implicit processing shows greatest differences between cannabis users and controls in centro-parietal sites whereas differences at fronto-central electrode sites are greatest for explicit processing. This presents as a task and scalp location reversal effect, where a reduced P3 for implicit processing at centro-parietal sites became enhanced at fronto-central sites. This fits with conclusions drawn by Krolak-Salmon et al that later ERP components are sensitive to attentional modulation of emotion that occurs as a result of top-down processing [25]. This revaluation of the face stimuli with top-down influence could be driving this reversal effect we observe. This gives a complex picture of cannabis effects on the P3 driven by attentional task demands. Early processing appears to be affected by cannabis exposure, reducing the P3, as is late processing in the empathic task condition. When attention is directed to emotion in the explicit condition we see a larger P3 in cannabis users compared to controls. What is not clear is how acute and residual effects might be driving this response pattern. The literature points to a THC dose dependent relationship [30] as well as one driven by acute compared to residual effects [31]. Recent research has looked at the role of cannabis exposure on early attention. Nichols et al used a flanker go/no go task to look at effects of early selective attention in cannabis users compared to controls. They showed a decrease in the N2 ERP associated with early selective attention in cannabis users compared to controls [46]. This is consistent with our data showing a decrease in the later P3 for implicit processing task which is most likely driven by early selective attentional processes. Our study however saw no differences behavioral measures whereas Nichols et al saw an increased reaction time response in cannabis users [46].

This suggests that consistent with previous research the effects of cannabis may be more closely tied to attention-based emotional processing rather than emotion processing that is occurring when attention is not directed towards a specific emotion [30, 31, 32]. Although in these studies the task was directed at visual or auditory attention whereas in our study we were targeting emotion processing. It seems likely though that cannabis affects one’s ability to consciously direct attention to specific elements of emotional stimuli.

This has implications for structural accounts of emotion processing [4]. Arguably, if emotion processing is limited to specific brain structures it is more likely that the effects of introducing an exogenous compound such as cannabis would affect a wider set of emotion processes irrespective of task. It seems that emotion processing then is less likely to be explained by single brain structures working independently but better explained by a distributed model where multiple sites are interacting with each other to fully process emotional stimuli [4]. It is also possible that cannabis effects these distributed brain areas differently which would suggest that possible distributions of cannabinoid receptors are playing a role in how cannabis modulates emotion processing.

#### Emotion

The P3 complex also appears to be modulated by the type of emotion being processed with negative emotions having a greater effect on P3 than positive and neutral emotions in our cannabis user group, an effect which is task dependent. This is consistent with the literature showing effects of cannabis on negative emotion [12], however it does not support structural imaging studies using behavioral tasks that show an increase in response to both negative and positive emotional stimuli [13]. Our behavioral data shows a larger behavioral rating response with negative emotions specifically in our empathy condition for both groups. Unlike previous research, we did not see a consistent increase in the P3 in relation to viewing emotional stimuli in all of our cannabis-using groups or across all variations of emotional stimuli [21, 22, 23, 24, 25]. Overall, there was an increase in the P3 for emotion in all conditions, however our data reflects a more complex pattern of emotional responses in brain activity driven by cannabis use. For example Rellecke et al showed that early ERPs were greater in participants viewing face stimuli expressing negative emotions in both implicit and explicit tasks but later components in the 200–600ms window were larger in those face stimuli expressing positive emotions [5]. It must be noted that Rellecke et al were not investigating the effects of cannabis use. Our data show emotional expressions compared to neutral stimuli elicited a reduced P3 in our cannabis user group compared to controls all scalp locations with the exception of centro-parietal sites. Responses in our control group were similar to those of Rellecke for the later time window consistent with our P3 [5].

As previously mentioned frequency of use appears to have an emotion specific effect. Although not significant, the reduction in P3 amplitude to fearful, happy and angry emotional expressions, is greatest in causal users compared to chronic users and controls. This is consistent with Ballard and colleagues who found the effects of cannabis use on emotion processing to be exacerbated for negative but not positive emotions [11]. Follow up studies making comparisons between casual and chronic use are needed to clarify this trend.

#### Empathy

Ability to empathize, measured as a behavioral response, appears consistent across groups for positive emotions. There was an expected reduction in ability to empathize with neutral expressions in all groups compared to positive and negative valence emotional faces. P3 effects did not reach statistical significance however there was a trend towards a greater P3 response for empathic responses to negative emotions compared to positive and neutral emotions in both groups. Further, while cannabis use was associated with differences in P3 during sex and emotion identification, empathic processing of the same facial expressions resulted in a reduction of these group differences. This supports previous discussion of the role of attention on emotion processing differences in the P3 for cannabis users [30, 31, 32] such that empathic responses draw on different processing mechanisms that are more emotionally-driven and less reliant on attention. Although not statistically significant, this effect was greatest in chronic users. Further research is needed to understand the relationship between cannabis use and task-driven emotion processing as related to empathic processing of facial expressions.

#### Limitations

One significant limitation of our study was not explicitly controlling for the acute, residual or long term effects of cannabis exposure although the majority of our cannabis users patterns of exposure best fit the residual and long term effects definition. Similarly the inability to control the amount and the type of cannabinoid our sample was exposed to makes our conclusions difficult to evaluate. This is both problematic and yet realistic as recreational cannabis users are unlikely to be able to access this information. Testing of recreational and medical cannabis in Colorado dispensaries for consumers is minimal if not nonexistent at this time. For example, THC content can range from 6–12%, to as high as 90%, in concentrates and edibles. We also did not control a priori for exposure to other substances such as alcohol and tobacco use. Although our sample reported minimal exposure to other substances, the residual effects of these substances are well documented to affect cognition, and could be contributing to our results. One particularly interesting point regarding our sample was that they were all using cannabis legally according to Colorado state law. It has become anecdotally apparent that this population tend to restrict their substance exposure to cannabis exclusively. However it should be acknowledged that this is a limitation of our study. As we develop a better understanding of the legal recreational cannabis industry we expect to be able to better address some of these questions.

## Conclusions

Our data show a significant effect of cannabis use on the P3 in an emotion processing task which was further modulated by task instruction. The P3 amplitude was reduced for negative emotions in our cannabis user group, compared to controls, and this effect was greatest when processing emotional expressions implicitly. There was a trend towards this being a dose dependent relationship with those users self-reporting the greatest exposure to cannabis having larger decrements in P3 amplitude. Attention driven demands on emotion processing appear to be affected by cannabis use as reflected in differences in the P3 amplitude.

## Appendix

The Recreational Cannabis Use Evaluation (R-CUE)

Cannabis Use Questionnaire:

1 Age: _________
2 Are you part of Colorado’s Medical Marijuana Registry (do you own a red card)?
a Yes
b No
3 If you answered yes to #2, how many years have you been a member of the registry?
a Less than one year (This is my first red card)
b 1–2 years
c 3–4 years
d 5–7 years
e 8–10 years
f 10+ years
4 How many years have you partaken in Cannabis use?
a Less than a year
b 1–2 years
c 3–5 years
d 6–10 years
e 11–15 years
f 16–20 years
g 20+ years
5 How many times a week do you use Cannabis (in any form)?
a Once a week: ______
b A couple of times a week: ______
c A few (3–6) times a week:______
d Daily: ______
e 2–4 times a day:_____
f More than 4 times a day:______
6 Which of the following ways do you like to intake cannabis, and which types of Cannabis do you prefer? Check all that apply (and check subcategories to the best of your knowledge/ability):
a Smoking Cannabis flower (Bud, Nugget, etc.): ______
i Indicas (“Body high”): _____
ii Sativas (“Mind high”): _____
iii Hybrids: ______
1 Sativa dominant hybrids: ______
2 Indica dominant hybrids: ______
3 True hybrids (50/50 of each): ______
b Smoking Cannabis Concentrates (Hashish/”Dabs”) ________
i Type of cannabis in concentrate:
1 Indica: _______
2 Sativa: _______
3 Hybrids: ______
a Sativa dominant hybrids: ______
b Indica dominant hybrids: ______
c True hybrids (50% of each): ______
4 Strain specific: _______
a If yes to strain specific hash, list strains that you have used: ________________________________________________________________________________________________________________________________________________
ii Method of THC extraction (the type of Concentrate) Check all that apply:
1 Solvent based extraction:
a Butane Honey Oil (BHO): ____
b Carbon Dioxide (CO2): ______
c Quick Wash Isopropyl Alcohol (QWISO): _____
d Hexane solvent concentrates: _____
e Propane solvent concentrates: ____
f Ethanol solvent concentrates: _____
g “Shatter” hash (High purity butane/ethanol extraction): _____
2 Solvent-less concentrates:
a Cold Water Extraction (CWE)/Icewax/Solvent-less wax/”grease”/”jewce”: _____
b Bubble hash: _____
c Screen filtered hash (Finger hash/Keif): _____
c Cannabis Edibles: _______
i Baked Edibles: _______
ii Hard Candy/ Gummy Edibles: ______
iii Chocolate Edibles: ____
iv Drink based edibles (THC infused sodas, teas, etc.): ______
v Tinctures: _____
1 Glycerin based: _____
2 Ethanol based: _____
vi Cannabis butter (Cannabutter): _____
vii Other (Please describe): ____________________________________________________________________________________________________________________________________________________________________________________
d Dermal Cannabis Application: ______
i Cannabis skin patches: _____
ii Cannabis lotions/balms/oils: ______
7 If you selected any of the flower/concentrate methods of cannabis intake, what smoking devices do you use? (Select all that apply)
a Water-filtration devices:
i Bong (upright/waterpipe):_____
ii Bong (gravity):_____
iii Bubbler: _____
b Dry smoking devices:
i Pipe (glass/metal): ______
ii Steamroller: ______
iii Joint: ______
iv Blunt:______
c Vaporizers:
i Bag vaporizers: ______
ii Whip vaporizers: ______
iii Portable/Pen vaporizers:________
d Dabs:
i Spoon dabs:_____
ii Nail dabs: ______
iii Noodle dabs: ______
iv Health stone dabs: _____
v Skillet dabs: _______
8 In order of preference (1 being most preferred, 4 being least preferred), what is your preferred form of consuming Cannabis: Cannabis flower/nugget, concentrates/hash, edibles, and topical absorption?
1 ____________________
2 ____________________
3 ____________________
4 ____________________
9 In order of preference (1-Most preferred, 10-Least preferred), which is you preferred method(s) of smoking/ingesting Cannabis?
1 _____________________
2 _____________________
3 _____________________
4 _____________________
5 _____________________
6 _____________________
7 _____________________
8 _____________________
9 _____________________
10 _____________________
10 In an average month, how much in Cannabis flower/nugget do you smoke?
a None:_____
b A gram or less: _______
c An eighth of an ounce (3.5 grams) or less:_____
d A quarter of an ounce (7 grams) or less:_____
e A half of an ounce (14 grams) or less:_____
f An ounce (28 grams) or less:_____
g More than an ounce: _____
h More than two ounces:_______
i More than a quarter pound (4 ounces):______
11 In an average month, how much in Cannabis concentrates do you smoke?
a None:______
b A gram or less: _______
c An eighth of an ounce (3.5 grams) or less:_____
d A quarter of an ounce (7 grams) or less:_____
e A half of an ounce (14 grams) or less:_____
f An ounce (28 grams) or less:_____
g More than an ounce: _____
12 In an average month, how many Cannabis edibles do you consume? (One edible is equal to what is considered one dose by the manufacturer).
a None:____
b One edible:____
c 2–4 edibles:______
d 4–8 edibles:______
e 10–20 edibles:______
f 30+ edibles:________

## Supporting Information

S1 Fig P3 Amplitude by Task and Emotion. (TIF)
