# Supplementary figures and images for: An Event-Related Potential Study on the Effects of Cannabis on Emotion Processing
Source: PLoS One. 2016 Feb 29;11(2):e0149764. doi: 10.1371/journal.pone.0149764 (PMC4772908; doi:10.1371/journal.pone.0149764)

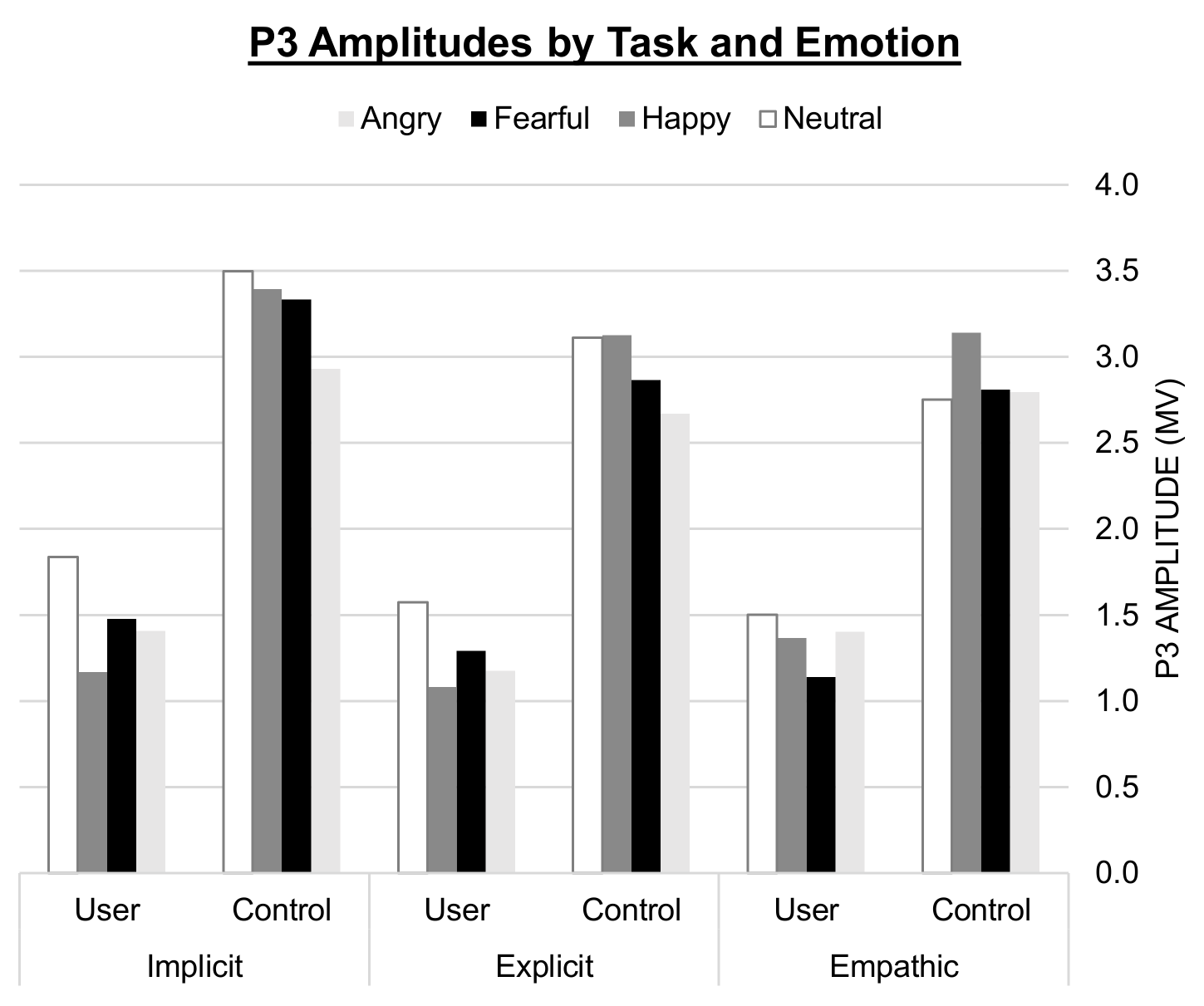

Supplement: S1 Fig — (TIF) [file pone.0149764.s001.tif]
